# “I Can Code”: An Innovative Workshop Introducing Students to Coding in the Context of Neuroscience Research

**DOI:** 10.59390/001c.160812

**Published:** 2026-05-19

**Authors:** Luca Iemi, Melissa Wright, Kara Pham, Anamaria Alexandrescu

**Affiliations:** 1 Neuroscience & Behavior Department Barnard College, New York, NY 10027, USA; 2 Center for Engaged Pedagogy Barnard College, New York, NY 10027, USA

**Keywords:** coding, programming, workshop, data analysis, contextualized learning, computational education, coding attitudes, student engagement, student confidence

## Abstract

Learning to code and teaching it present distinct challenges for both students and instructors. This paper describes a workshop designed to help instructors introduce coding to neuroscience undergraduates and to help students get started with coding. The workshop integrates learning within a neuroscience context: instead of using abstract examples, students apply coding directly to real data from a published neuroscience research paper. The workshop was delivered as a brief (2–3-hour) session embedded within a required undergraduate neuroscience laboratory course. Survey data showed the workshop significantly enhanced students’ learning experience, boosting enjoyment, confidence, self-rated coding ability, and perceived usefulness. These findings suggest that the workshop can enhance students’ motivation, leading to better engagement and learning outcomes. We propose that this model of contextualized learning could help overcome current barriers in computational education for neuroscience.

As neuroscience research generates increasingly large and complex datasets through techniques such as neuroimaging, electrophysiology, and genomics, computational tools have become essential for data analysis and interpretation. Preparing students for this evolving research landscape requires strong quantitative foundations, including statistics and experimental design, as well as practical programming skills (Akil et al., 2016; Goldman & Fee, 2017; Grisham et al., 2017; Juavinett, 2022). Additionally, coding skills are now widely expected across diverse industries and often enhance employability and earning potential. Accordingly, teaching coding is not only a matter of skill development but also an opportunity to advance equity, giving the diverse population of undergraduate students the tools to succeed in both academia and other professional careers (Juavinett, 2022). Despite the clear importance of coding skills for students, courses that teach coding are rarely included in neuroscience degree programs. A recent survey found that very few schools offered required or elective computer science courses for the neuroscience major (Pinard-Welyczko et al., 2017). This highlights a significant gap in undergraduate training, leaving most neuroscience students without formal opportunities to develop essential computational skills at the undergraduate level.

Even when coding courses are offered, instructors often face challenges in determining the most effective ways to teach, particularly in introductory courses, due to several barriers. First, many instructors have limited expertise in coding and may therefore be unfamiliar with how to teach coding effectively. This is supported by a recent survey (Juavinett, 2022) which found that fewer than half of neuroscience faculty had taken a coding class or felt comfortable working with code. Second, students often find learning to code difficult, as reflected in the high dropout rates reported in introductory programming courses (Bennedsen & Caspersen, 2019). Moreover, the social and psychological context of introductory courses adds another layer of complexity for instructors and students alike. For many first-year students, these courses function not only as an introduction to a discipline, but also as a test of their overall fit in college, creating high-pressure situations that can affect their interest, motivation, and, in turn, their learning experience (Harackiewicz et al., 2016). These pressures are particularly pronounced for student subgroups who have been historically (and continue to be) underrepresented in coding, such as women, racial and ethnic minorities, and first-generation students (Fabiano, 2022; Holloman et al., 2021; Lewis et al., 2016). Such students may already doubt their belonging and competence in academia, increasing the likelihood of disengagement or dropout. Consequently, instructors must design courses that not only teach coding effectively but also support students’ sense of inclusion and confidence. Addressing these challenges is critical for the success of both instructors and students in introductory coding courses. To this end, in this paper, we introduce an innovative coding workshop specifically designed for students with no prior coding experience and present survey data on its impact on students’ learning experience and attitudes toward coding. The workshop was delivered as a brief (2–3-hour) session embedded within a required undergraduate neuroscience laboratory course.

This workshop is grounded in a contextual learning approach, which consists of acquiring new knowledge through discipline-specific problems rather than abstract exercises. Note that “context” refers to the specific domain in which students apply their skills—in this case, neuroscience. In educational research, context has been shown to shape the perceived value of a task: when students perceive learning materials as relevant to their studies and future goals, they are more motivated, engaged, and successful (biology education: Allen & Tanner, 2003; chemistry education: Vaino et al., 2012). Accordingly, our workshop integrates learning to code within a neuroscience context: instead of practicing with abstract variables and computations, students apply coding directly to real data from a neuroscience research paper. Importantly, research suggests that contextualized coding classes can improve attitudes toward programming and encourage participation from students who don’t see themselves as ‘computational’ and might otherwise avoid standard courses. These classes are also more effective at recruiting students who are traditionally underrepresented, such as women and students of color (Zuckerman & Juavinett, 2024). Accordingly, this approach not only boosts students’ motivation and academic success but also promotes equity in computational education.

While introducing coding into the undergraduate neuroscience curriculum presents many challenges, this study demonstrates how a contextualized learning approach can make the process more engaging and effective for both students and instructors.

## MATERIALS AND METHODS

### Participants

A total of 63 female undergraduate students participated in this study during the Spring 2025 semester, reflecting the student body of the women’s college where the study was conducted. The mean age of the students was 19.3 years (SEM = 0.15, range: 17-23). All were enrolled in one of six sections of *Laboratory in Neuroscience*, an intermediate-level course required for the major offered by the Department of Neuroscience and Behavior at Barnard College (Columbia University, New York City). This introductory laboratory course meets once a week for 4 hours and consists of three modules: neuroanatomy, histology, and electrophysiology, which introduce students to these experimental methods through hands-on experimental design, execution, and analysis. Enrollment in this course requires prior completion of the lecture course *Introduction to Neuroscience*, which introduces students to the anatomy and physiology of the nervous system, including neural structure and function, neurotransmission, sensory and motor systems, cognition, and clinical applications. No coding skills are required to enroll in the laboratory course.

To teach students how to apply experimental design and data analysis to the laboratory experiments, we introduced them to basic experimental design and statistics concepts and to data analysis and visualization using MATLAB. The coding component of the course began with the coding workshop described here, which students were required to complete as part of the course requirements. Two anonymous surveys, graded based on completion, were administered to understand the impact of the coding workshop on student’s attitudes toward coding. Because the analysis required matching responses across the two surveys from the same individuals, 11 participants were excluded due to unmatched or missing data. The final sample included 52 participants. The study protocol was approved by the Institutional Review Board for research with human participants prior to data collection.

### Methods of the Coding Workshop

The coding workshop was conducted during one class session. Students completed the in-class workshop at their own pace, with the majority of students finishing it in 2–3 hours, while a minority of students spent the entire class duration of 4 hours working on it.

#### Pre-class Assignments

Before the in-class workshop, students completed the free MATLAB Onramp online tutorial (~2 hours: https://matlabacademy.mathworks.com/details/matlab-onramp/gettingstarted), which introduces MATLAB basics through self-paced interactive lessons run in the web browser. Students accessed the tutorial via the MathWorks website using their institutional email and submitted a PDF certificate of completion on Canvas. After completing the tutorial, students were required to install the MATLAB version R2022a or set up access to MATLAB Online using their institutional email. This ensured all students arrived to class with working access to MATLAB and basic familiarity with the interface and core functions. Students were also assigned a pre-workshop homework assignment (~3 hours), which consisted of: completing a pre-survey, reading a short statistics and experimental design guide, reading Spanò et al. (2020) (i.e., a research paper examining the role of the hippocampus in dreaming by comparing dream reports from individuals with bilateral hippocampal damage and healthy controls), and completing a Canvas quiz. The statistics and experimental design guide introduced students to basic concepts of experimental design such as experimental variables, conditions, and subject designs, and of descriptive and inferential statistics such as measures of central tendency and variability, statistical significance, and common statistical tests. The Canvas quiz consisted of multiple-choice questions about these statistics and experimental design concepts applied to the assigned paper. These materials are provided in the Supplementary Materials.

#### In-class Workshop

The in-class portion of the coding workshop was designed to introduce students to data analysis and visualization in MATLAB using the data from a research paper by Spanò et al. (2020). We selected this study because it features straightforward data analysis, clear scientific writing, accessible data, effective visualizations, and a research topic (i.e., dreaming) that is engaging for undergraduate students. The workshop integrated structured MATLAB exercises with conceptual questions tied directly to the study’s methods and findings. Students began by reviewing basic MATLAB operations, including variable assignment, arithmetic operations, and matrix manipulation, using numerical values reported in the article’s data tables. These exercises were intended to develop fluency with arrays, matrices, and tables in MATLAB. For instance, students calculated hippocampal volume loss in individual patients relative to controls and created matrices representing participants’ ages and disease chronicity to practice indexing and array construction. Next, students imported data from Spanò et al. (2020) into MATLAB (i.e., Table 2-Source data) and performed a between-subjects analysis to test one of the study’s main hypotheses that individuals with hippocampal damage experience fewer dreams than healthy controls. Students wrote MATLAB code to extract dream frequency data for each group, compute descriptive statistics (mean, standard deviation, standard error), and perform a Mann-Whitney U test to assess statistical significance. They then visualized the results with bar plots, complete with error bars and customized axes and labels. Students were asked to compare their plots to Figure 1D in the article to verify the accuracy of their analysis and interpret the implications of their findings. The findings revealed that dream frequency was lower in patients compared to controls, in line with the hypothesis that the hippocampus is necessary for typical dreaming. To promote critical thinking, students conducted a novel within-subject analysis not included in the original study. This analysis tested a common assumption: that dreams are more frequent during REM sleep than during NREM sleep. Unlike the previous between-group analysis, this task required students to combine data across both groups and compare two variables—REM and NREM dream proportions—within each participant. Students extracted the relevant data from the table, calculated the mean, standard deviation, and standard error for each sleep stage, and performed a paired-sample t-test to assess significance. They then visualized their results using a bar chart and interpreted the outcome, which—contrary to the assumption—showed no significant difference in dream frequency between REM and NREM sleep. Throughout the workshop, students were encouraged to use the MATLAB documentation (*Help*), check their outputs against published results in the manuscript, and annotate their scripts to reinforce good coding practices. The exercises were designed to progressively build confidence in coding while deepening students’ understanding of experimental design, statistical analysis, and data visualization in the context of cognitive neuroscience.

**Figure 1.**
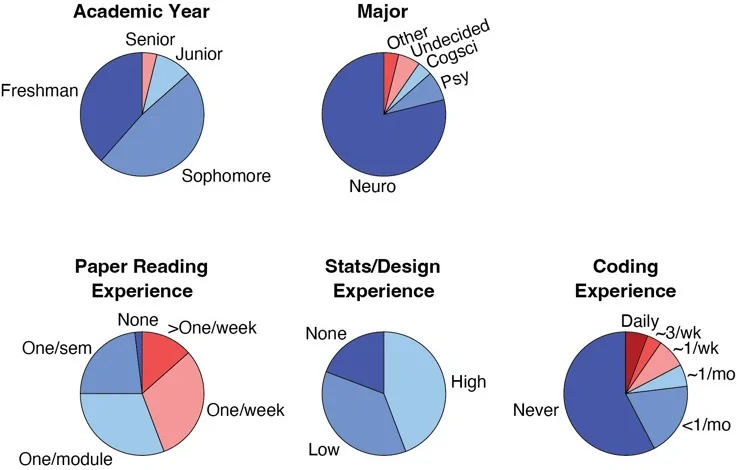
Student Sample Information. Pie charts show the distribution of the student sample (N=52) across academic year, major, and self-reported prior experience with core academic skills, including reading scientific papers, statistics/experimental design, and coding.

The in-class workshop was graded using a rubric to promote student engagement. Students submitted completed MATLAB Live Scripts, and the required figures and captions (see instructions at the end of the provided MATLAB Live Script). A grading rubric and answer key for these submissions are available upon request.

The MATLAB Live Script used in the in-class workshop (CodingWorkshop_JUNE.mlx) is available in the Supplementary Materials.

### Methods of the Survey

#### Survey Data Collection

We assessed changes in students’ attitudes toward coding using a pre–post survey design. Before the workshop, students completed a pre-survey at home to report their baseline attitudes toward coding. At the end of the class session, they completed a post-survey to report their attitudes and the perceived impact of the workshop. Both surveys were administered through Canvas as ungraded surveys, with no time limit and only one attempt. Submissions were anonymous.

The pre-survey included demographic and background questions, such as students’ academic year, intended or declared major, the average number of peer-reviewed research articles read as part of coursework or research (Hubbard & Dunbar, 2017), frequency of using experimental design and statistics in academic or research settings, frequency of coding, and coding languages used.

Both the pre- and post-surveys included 6 items assessing attitudes toward coding. Five (#1–5) were adapted from the Elementary Student Coding Attitudes Survey developed by Mason and Rich (2020) and one (#6) was newly added for this study. Students rated their agreement with each item on a 6-point Likert scale (‘Strongly Agree’, ‘Agree’, ‘Somewhat Agree’, ‘Somewhat Disagree’, ‘Disagree’, ‘Strongly Disagree’). The items were:

1. I like coding, or I think I would like coding.
2. I can learn to code.
3. I am good at coding.
4. I can use coding skills in other school subjects or in my research.
5. I think I will need to understand coding for my future job.
6. I am interested in applying coding to neuroscience data.

The post-survey included the 6 core attitude items as well as four additional items: three assessing the workshop’s impact on a 6-point scale (from “Very positive impact” to “Very negative impact”) on coding interest and understanding, and one comparing the learning effectiveness of the in-class workshop to the effectiveness of the MATLAB Onramp online tutorial on a 6-point scale ranging from strongly favoring the in-class session (“The in-class workshop was much more effective”) to strongly favoring the online tutorial (“The online tutorial was much more effective”). These items were:

- What impact has the in-class workshop had on your interest in using coding?
- What impact has analyzing data from a neuroscience research article (Spanò et al., 2020) had on your interest in coding?
- What impact has the in-class workshop had on your understanding of coding?
- In terms of your learning, how did the in-class workshop compare to the at-home online MATLAB tutorial?

Additionally, the post-survey included three open-ended questions asking for qualitative feedback on the workshop:

- Reflecting on your experience of the in-class MATLAB workshop, state one aspect of the workshop that supported your learning. Explain.
- Reflecting on your experience of the in-class MATLAB workshop, state one aspect of the workshop that hindered your learning. Explain.
- Please feel free to share any additional suggestions for future iterations of the coding module of this course.

### Quantitative Analysis

We evaluated changes in students’ pre- and post-workshop attitudes (Fig. 2) using paired t-tests. Responses were coded from –2.5 (“Strongly disagree”) to +2.5 (“Strongly agree”). We considered a #change significant if the p-value <0.008, applying Bonferroni correction for multiple comparisons (0.05/6 tests).

**Figure 2.**
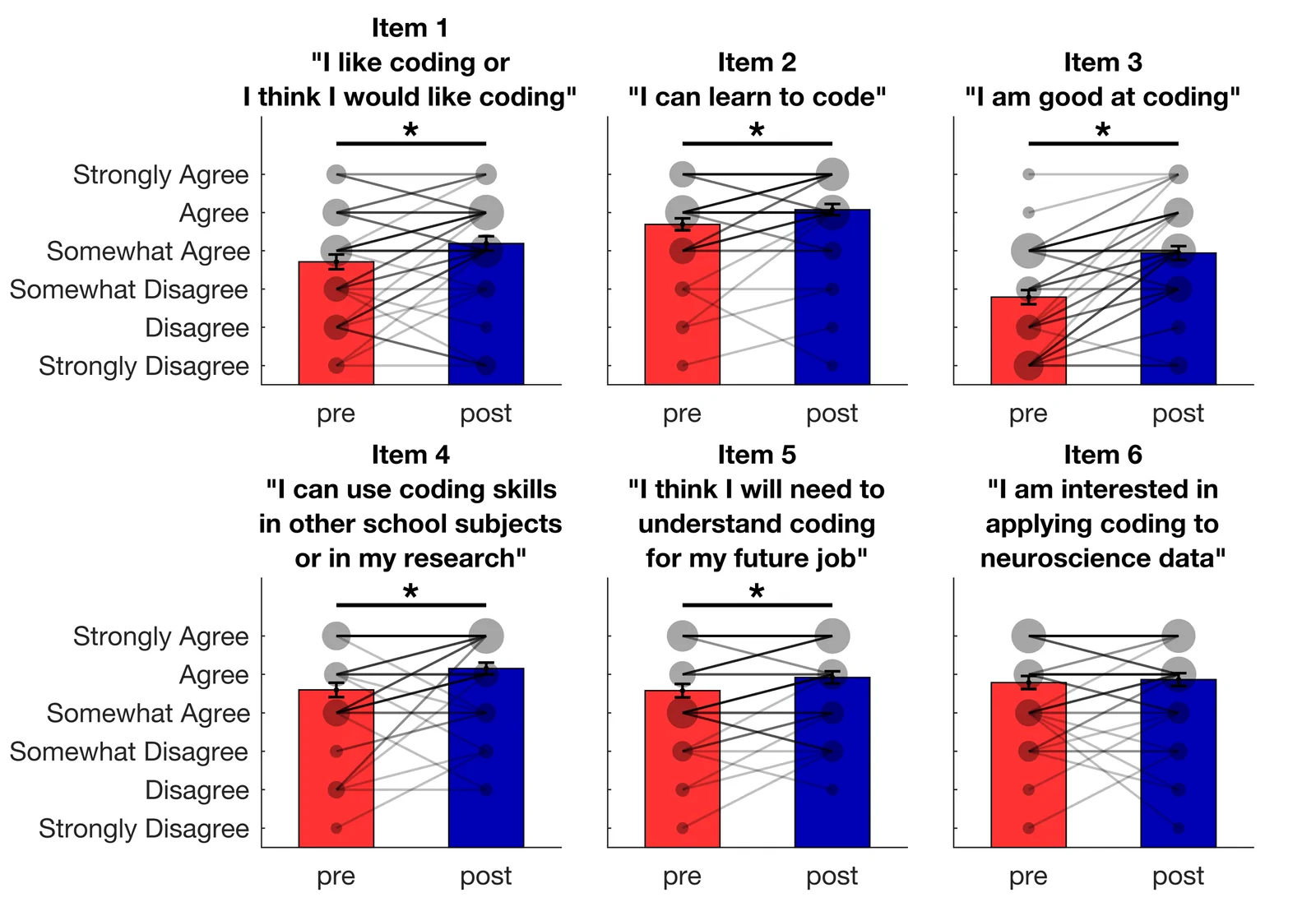
Changes in self-reported coding attitudes following the workshop. Students rated their agreement with six statements (Items 1–6) on a 6-point Likert scale before (pre) and after (post) the workshop. Responses were coded to range from –2.5 (“strongly disagree”) to +2.5 (“strongly agree”). Bars indicate mean ± standard error of the mean (SEM). Asterisks indicate statistically significant pre-post differences (paired t-test, p < .008, Bonferroni corrected). Circles indicate participant counts (marker size proportional to count). Lines connect paired pre- and post-responses for each participant (line darkness proportional to the number of participants showing the same change).

To evaluate whether the workshop’s impact varied across student demographics and prior experience, we conducted a between-subjects analysis. We computed two composite scores: a composite impact score, representing the perceived benefit of the workshop (sum of the ratings for its impact on coding interest, coding understanding, and the impact of analyzing neuroscience data on interest), and a composite attitude score, representing overall attitudes toward coding (sum of the six core attitude items). We then tested for differences in these composite scores between subgroups defined by pre-workshop survey data. These subgroups included academic year (freshman vs. non-freshman), major (neuroscience vs. other), prior coding experience (novice [none] vs. experienced [any frequency]), experience reading scientific papers (novice [none or one per semester] vs. experienced [one per module or more]), and experience with statistics/experimental design (novice [never, in one course, or occasionally in research] vs. experienced [in several courses or often in research]). Group differences were assessed using independent-samples t-tests (freshman - non-freshman; neuroscience – other; novice- experienced). We considered a change significant if the p-value <0.005, applying Bonferroni correction for multiple comparisons (0.05/10 tests).

All statistical analyses were performed using MATLAB (R2022b; The MathWorks; RRID:SCR_001622).

#### Qualitative Analysis

Open-ended responses were analyzed using an inductive thematic analysis approach to explore how different aspects of the instructional design influenced students’ learning experience, both positively and negatively. Responses were reviewed and coded manually by the research team to identify recurring patterns related to factors supporting or hindering learning.

## RESULTS

### Student Sample Information

The student sample was composed of Freshmen (n = 20), Sophomores (n = 25), Juniors (n = 5), and Seniors (n = 2). The majority of students were neuroscience majors (n = 41), with the rest distributed across other disciplines, including psychology (n = 4), cognitive science (n = 2) and other disciplines (n = 2), or they were undecided at the time of the survey (n = 3). Prior experience with reading scientific literature was common among students. The frequency with which students read scientific papers was distributed as follows: one paper per semester (n = 12), one per course module (n = 16), one per week (n = 16), and more than one per week (n = 7), with only one participant having no prior experience. Prior experience with statistics was also common among students. The majority of students reported having had some experience in statistics, either in one course or occasionally in their research (Low experience; n = 19), or in multiple courses or often in their research (High experience; n = 23). A minority of students reported having no prior experience (n = 10). Prior to the workshop, most participants had little to no coding experience. More than half of the sample reported they had never coded (n = 30). Of those with some experience, the frequency of coding was generally low: less than once a month (n = 10), about once a month (n = 3), about once a week (n = 4), about 3 times a week (n = 2), and almost every day (n = 3). Of the 22 participants who did have coding experience, only 2 specified using MATLAB. The remaining 20 participants reported experience with other coding languages (i.e., Python, R, Java, C++).

### Pre- to Post-Workshop Changes in Coding Attitude

A comparison of post- vs. pre-workshop survey data revealed significant improvements in students’ attitudes toward coding (Fig. 2). Ratings were collected on 6-point Likert scales from –2.5 (“strongly disagree”) to +2.5 (“strongly agree”). Relative to pre-workshop, post-workshop students reported significant increases in enjoyment of coding (Fig. 2 top left panel), confidence in learning to code (Fig. 2 top middle panel), self-rated coding ability (Fig. 2 top right), and perceived usefulness of coding for school or research (Fig. 2 bottom left panel) and for jobs (Fig. 2 bottom middle panel). Finally, interest in applying coding to neuroscience data did not change significantly (Fig. 2 bottom right panel). Table 1 summarizes the descriptive and inferential statistics of the pre- vs. post-workshop comparisons.

**Table 1.** Pre- to Post-Workshop Changes in Coding Attitudes.

**Survey Item**	**Pre Rating** **(mean ± SEM)**	**Post Rating (mean ± SEM)**	**t(51)**	**p-value**	**Mean of differences 95% CI**	**Cohen's d**
“I like coding, or I think I would like coding.”	0.21 ± 0.19	0.69 ± 0.19	2.939	0.004 (*)	[0.15, 0.81]	0.41
“I can learn to code.”	1.19 ± 0.16	1.58 ± 0.15	3.045	0.0037 (*)	[0.13, 0.64]	0.42
“I am good at coding.”	–⁠0.71 ± 0.18	0.44 ± 0.18	6.966	< 0.0001 (*)	[0.82, 1.49]	0.97
“I can use coding skills in other school subjects or in my research.”	1.10 ± 0.19	1.65 ± 0.15	3.365	0.0015 (*)	[0.23, 0.89]	0.47
“I think I will need to understand coding for my future job.”	1.08 ± 0.17	1.42 ± 0.16	3.163	0.0026 (*)	[0.13, 0.57]	0.44
“I am interested in applying coding to neuroscience data.”	1.29 ± 0.17	1.37 ± 0.17	0.562	0.5766 (n.s.)	[-0.20, 0.35]	0.08

Summary of descriptive and inferential statistics of the comparison of ratings of students’ self-reported attitudes toward coding before and after the workshop. Asterisks denote statistically significant changes (paired t-test, *p < 0.008, Bonferroni corrected).

### Workshop’s Perceived Impact

Students reported a positive experience of the workshop. Because this analysis aimed to characterize students’ perceived experience, results are presented using descriptive statistics. Specifically, 92.3% and 96.2% of students reported that the in-class workshop had a positive impact on their coding interest and understanding, respectively (Fig. 3 top panel). Moreover, 88.5% of students reported that the experience of analyzing neuroscience data from a research paper positively impacted their coding interest (Fig. 3 top panel). Finally, 78.8% of students rated the in-class workshop as more effective compared to the online tutorial that was part of the pre-class assignments (Fig. 3 bottom panel).

**Figure 3.**
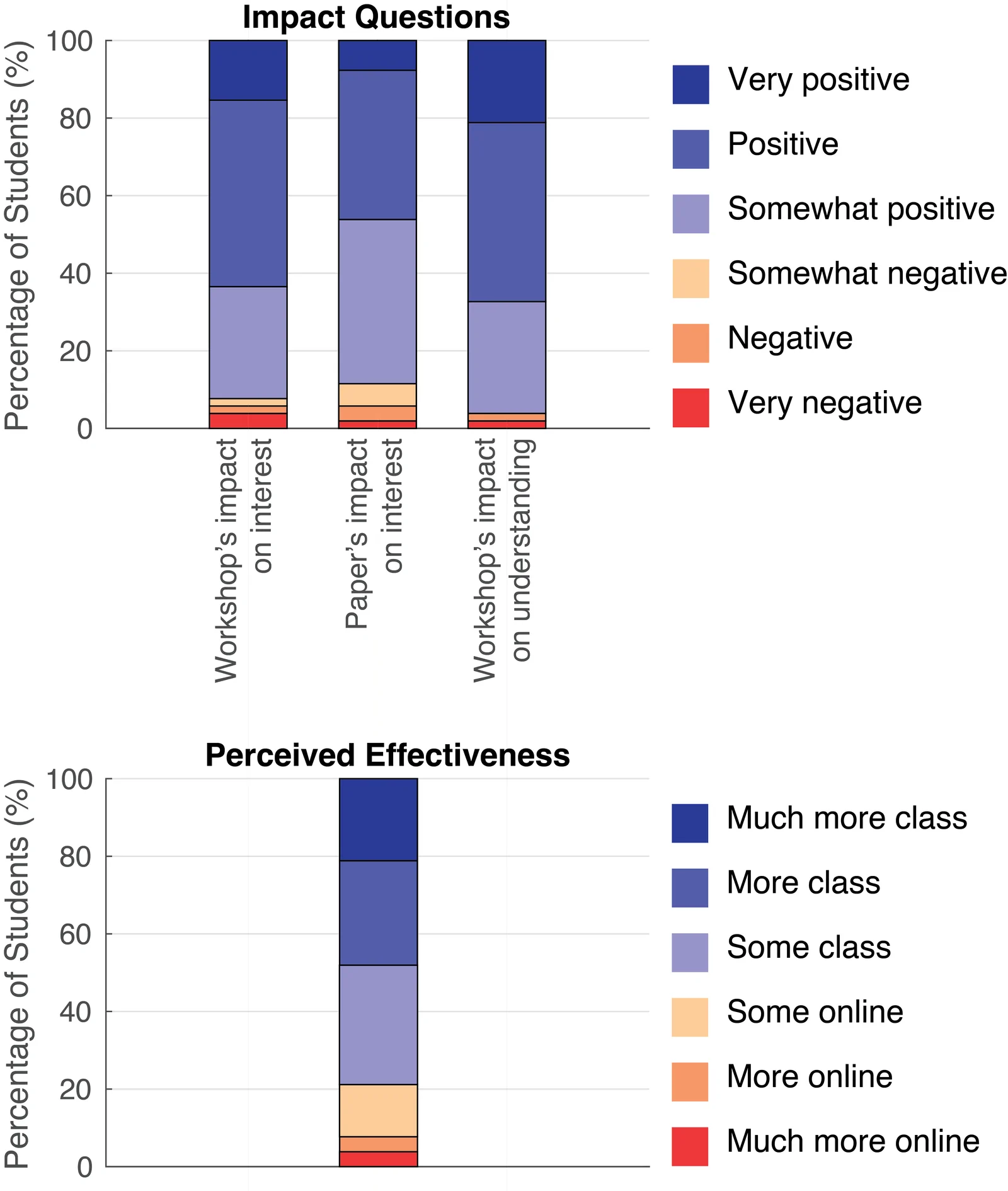
Student perceptions of the workshop’s impact and effectiveness. Bars represent the percentage of students selecting a response for each survey item on a 6-point scale. Top panel: distributions of ratings for (1) the in-class workshop’s impact on interest in coding, (2) the impact of analyzing data from the research paper on interest in coding, and (3) the in-class workshop’s impact on understanding of coding (scale from –2.5 = *very negative impact* to +2.5 = *very positive impact*). Bottom panel: ratings comparing the perceived learning effectiveness of the in-class workshop versus the at-home MATLAB Onramp tutorial (–2.5 = *online tutorial much more effective* to +2.5 = *in-class workshop much more effective*). Colors correspond to response categories (from most negative to most positive).

### Interaction Between Workshop Experience and Student Backgrounds

Between-subject analysis revealed no significant differences across student subgroups defined by year, major, paper reading experience, statistics experience, or coding experience at either uncorrected or Bonferroni-corrected significance thresholds. We combined survey items into composite scores that summarized overall attitudes toward coding and the perceived impact of the workshop. Differences in these composite scores between subgroups were assessed using independent-samples t-tests. No significant group differences were observed in the composite attitude score (year: t(50) = 1.281, p = 0.206; major: t(50) = 0.334, p = 0.740; paper experience: t(50) = 0.236, p = 0.814; statistics experience: t(50) = –1.246, p = 0.219; coding experience: t(50) = 0.553, p = 0.582) or in the composite impact score (year: t(50) = 0.921, p = 0.361; major: t(50) = -0.594, p = 0.555; paper experience: t(50) = -1.820, p = 0.075; statistics experience: t(50) = -1.987, p = 0.052; coding experience: t(50) = -0.839, p = 0.405).

### Thematic Analysis of Open-Ended Responses

A thematic analysis of student feedback revealed distinct themes regarding aspects that both supported and hindered student learning. Four primary themes emerged characterizing elements that successfully supported learning:

1. Immediate instructional support: Many students emphasized that having instructors and teaching assistants available in real time allowed them to quickly resolve coding issues that would otherwise have been discouraging when working alone, and that peer collaboration further supported troubleshooting and understanding. (Based on multiple responses such as “opportunity to ask for help… as opposed to doing it by myself online”, “having professor/TA nearby”, “working with peers helped talk through questions.”)
2. Applied context: Students frequently noted that working with real neuroscience research data increased engagement and helped them feel like active participants in the research process, while comparing their outputs with published figures reassured them that their analyses were correct. (Based on responses such as “felt like a real researcher”, “using actual data from a research article helped apply coding knowledge,” “checking work against the paper reduced anxiety.”)
3. Scaffolded pedagogical design: Several students highlighted that the step-by-step instructions helped them gradually build independence, allowing them to troubleshoot errors using documentation and notes rather than relying solely on instructor assistance. (Based on *“very step-by-step,” “clear instructions,” “encouraged consulting MATLAB documentation,” “smooth transition into more complicated tasks.”*)
4. Active engagement: Students also described learning through experimentation and error correction, noting that generating figures and debugging mistakes helped consolidate both conceptual understanding and coding skills. (Based on “made mistakes and learned from them,” “creating graphs helped me understand,” “actively working through problems helped me learn techniques.”)

Analysis also revealed three principal themes representing obstacles to learning:

1. Technical challenges: Students reported that software installation issues, file uploads, slow loading times, and occasional MATLAB malfunctions diverted attention away from learning coding concepts and required substantial troubleshooting time. (Based on toolbox download problems, MATLAB navigation difficulties, WiFi crashes, loading delays.)
2. Time pressure: Many students indicated that the fixed class duration created a sense of urgency, particularly for those with no prior coding experience, leading some to feel rushed or fatigued during the extended period of screen-based work. (Based on “felt rushed,” “not enough time,” “too long in one sitting,” “worried about others finishing first.”)
3. Instructional quality: Some students reported moments of procedural confusion, describing difficulty understanding the rationale behind certain coding steps or feeling that parts of the instructions assumed background knowledge they did not yet possess. (Based on “felt like following steps without understanding why,” “needed prior knowledge,” “instructions unclear.”)

### DISCUSSION

Learning to code and teaching it present distinct challenges for students as well as instructors. In this paper, we present an innovative workshop designed to help instructors introduce coding to neuroscience undergraduates and to help students get started with coding. By integrating the learning process in a neuroscience context, the workshop aimed to build technical skills while also supporting student motivation.

Using survey data, we found that the in-class workshop had a positive effect on students’ attitudes toward coding and was perceived as impactful. Consistent with our predictions based on contextualized learning, the workshop supported three key dimensions of student motivation: interest, self-efficacy, and perceived usefulness. First, it increased students’ interest in coding. Specifically, the experience of working with data from a research paper was reported as particularly effective in making coding feel interesting, demonstrating the value of contextualization. We believe that, by embedding coding within authentic neuroscience problems—such as testing hypotheses about the neural correlates of dreaming—students were able to channel their pre-existing curiosity in neuroscience directly into computational practice. This is particularly relevant pedagogically as increased interest promotes academic achievement (Harackiewicz et al., 2016; Hidi & Harackiewicz, 2000) and fosters intrinsic motivation, which has been shown to facilitate learning of both intended and incidental material (Gruber et al., 2014; Kidd & Hayden, 2015). Second, the workshop enhanced students’ coding self-efficacy, or confidence in their ability to succeed in coding tasks. This was likely achieved through scaffolding: students initially followed structured steps and then gradually worked more independently, while receiving ongoing feedback from comparing their results to published findings and occasionally from the teaching team. This approach allowed students to successfully complete meaningful tasks—so-called mastery experiences—which are known to strengthen confidence and self-efficacy in learning new skills (Belland et al., 2013). Third, the workshop increased students’ perceptions of coding as a useful tool for both academic and professional purposes. Using a contextualized approach, students took on the role of scientists and could directly see the relevance of what they were learning. Recognizing the value of their work is known to enhance students’ motivation, engagement, and performance (Allen & Tanner, 2003; Vaino et al., 2012). Interestingly, we did not find a significant change in students’ interest in applying coding to neuroscience data. A likely explanation is a ceiling effect: this item received the highest baseline ratings of all attitude measures, suggesting that students already valued coding as a tool for neuroscience prior to the workshop. Notably, this item was not part of the original Elementary Student Coding Attitudes Survey (Mason & Rich, 2020), so it is not possible to compare this finding to previous research. In summary, the results of the survey suggest that contextualized coding instruction can foster interest, self-efficacy, and perceived usefulness—three factors that pedagogical research indicates support learning through boosting motivation.

Students reported that the contextualized coding workshop was more effective than the online tutorial, which relied on abstract exercises unrelated to neuroscience. This positive result may be due to two main factors. First, the sequential order (completing the on-line tutorial before the in-class workshop) likely prepared students for the hands-on session, enhancing self-reported effectiveness. Second, it could be an effect of contextualized learning, consistent with our prediction.

We also examined whether the workshop had differential effects across students’ subgroups. No significant differences were observed across subgroups defined by major, academic year, or experience in core academic skills. This could reflect limited statistical power given the modest sample size, but it may also suggest that the workshop is broadly effective across a wide range of student backgrounds.

Our findings contribute to the ongoing discussion about the future of computational education. With the rapid development of artificial intelligence (AI) tools, educators must reconsider how coding is taught at the undergraduate level (Giannakos et al., 2025). Although our workshop did not use AI, the approach we present could be extended in several ways. For example, AI could generate coding tasks from other published neuroscience datasets, which could be tailored to students’ individual interests. This context-personalization strategy may further enhance engagement and intrinsic motivation beyond the levels observed in our current approach (Harackiewicz et al., 2016). Second, AI could offer adaptive scaffolding and interactive support to guide students as they practice programming concepts. Developing the ability to collaborate with AI tools while critically evaluating their output may become an essential part of future computational education.

It is important to note that the workshop was intentionally designed to introduce students to scripting practices, such as writing sequences of commands to import, analyze, and visualize data, as an accessible entry point into computational thinking for students with no prior experience. Although similar tasks can be completed using software that does not require scripting, we believe that learning to script is critical for developing more advanced coding skills as students gain experience. Future iterations could extend this contextualized approach to more advanced coding workshops.

Several limitations of our workshop should be noted. First, the choice of MATLAB was motivated by the expertise of the teaching team. However, MATLAB is costly without an institutional license, and software installation can create a logistical barrier for students. By contrast, Python and R are free, widely used, and increasingly prevalent in neuroscience and data science (e.g., Neuromatch Academy). Future iterations of the workshop could explore implementation using these other coding languages to broaden accessibility. Second, qualitative feedback revealed pedagogical challenges. Students cited technical issues with the programming software, time pressure from the workshop’s length, and occasional ambiguity in instructions. Addressing these challenges will be essential for future iterations—for example, by splitting the workshop into shorter sessions, simplifying instructions, or providing guidance for handling technical issues.

An additional limitation is the reliance on self-reported survey measures. Although positive attitudes toward coding may support academic performance, they do not necessarily translate into mastery of coding skills. Accordingly, these findings should not be interpreted as evidence that the workshop optimizes learning outcomes. Future work should incorporate performance-based assessments, such as objective measures of coding proficiency.

In conclusion, the workshop we present here offers an effective way to introduce coding to undergraduate students. Our survey results suggest that teaching coding with research data can enhance the learning experience of undergraduate neuroscience students. While this workshop is just one example, it demonstrates how contextualized learning can support skill development and reduce barriers for both instructors and students in computational education relevant to neuroscience research.

### Address correspondence to:

Dr. Luca Iemi, Neuroscience & Behavior Department, Barnard College, New York, NY 10027, USA. Email: liemi@barnard.edu

## Supplementary Material

Coding Workshop Instructions

Canvas Quiz

Experimental Design and Statistics Guide
