# Supplementary material for: “I Can Code”: An Innovative Workshop Introducing Students to Coding in the Context of Neuroscience Research
Source: J Undergrad Neurosci Educ. 2026 May 19;24(2):51–60. doi: 10.59390/001c.160812 (PMC13625906; doi:10.59390/001c.160812)
Supplement: Supplementary file 1 [file junejournal_2026_24_2_160812_340611.pdf]

As part of the**Coding Workshop**, you will use the instructions below to analyze and plot the data collected by Spano et al (2020).

Please read all of the information and follow the directions carefully.

To execute specific sections of the code in MATLAB, simply click within the desired gray section (Live Editor Box), section, and then select "Run Section" located near the top of the MATLAB window. Alternatively, you can run the entire script by clicking the "Run" option on top of the MATLAB window.

1. MATLAB Basics

Variables

- Variables store information such as numbers or text.
- A variable can store one value or multiple values in an array or matrix.
- Each stored value is referred to as an element.
- Variable names must start with a letter and contain only letters, numbers, and underscores. When possible, variable names should be descriptive.
- When you run your MATLAB code, the variables you create will appear in the "workspace".

Example: Assign the value, 2, to the variable, x.

- `x = 2;`

Basic arithmetic operations

- You can perform basic math operations using numbers and/or variables.
- To perform element-wise addition, subtraction, multiplication, and division, you can use these operators: "+", "-", ".*", "./".
- Keep the order of operations (PEMDAS) in mind when writing equations.
- More information can be found here: MathWorks Arithmetic Operations

Example: Multiply the variable, x, by 4 and assign the output to the variable, y.

- `y = x.*4;`

Using the values in table 1 in Spano et al. (2020), practice working with MATLAB variables by writing the code below:

1. Assign the LHPC volume for patient HPC1 to new variable, "vol1".

```
%Your Code Here. Will require 1 line of code.
```

2. Assign the average LHPC volume for control subjects to a new variable, "cntrl".

```
%Your Code Here. Will require 1 line of code.
```

3. Calculate the difference in LHPC volume between patient HPC1 and the average LHPC volume of control subjects by subtracting "cntrl" from "vol1" and assign it to a new variable, “diff1”.

```
%Your Code Here. Will require 1 line of code.
```

4. Calculate the percentage LHPC volume loss of patient HPC1 relative to the average volume in controls and assign it to a new variable, "loss1". To calculate "loss1", divide "diff1" by "cntrl" and then multiply the result by 100.

```
%Your Code Here. Will require 1 line of code.
```

5. Calculate the percentage LHPC volume loss of patient HPC2 relative to the average volume in controls and assign it to a new variable, "loss2".

```
%Your Code Here. Will require ~1-3 lines of code.
```

6. Calculate the percentage LHPC volume loss of patient HPC3 relative to the average volume in controls and assign it to a new variable, "loss3".

```
%Your Code Here. Will require ~1-3 lines of code
```

If the results match those in Table 1 in Spano et al, (2020), you can move to the next section. If they do not, ask your instructor or TA for help.

2. Arrays and Matrices

Array Creation

All MATLAB variables are multidimensional arrays. Even a variable with a single element is considered to be a 1x1 array, i.e an array with 1 row and 1 column. Matrices are a type of array that contains two dimensions, rows and columns. A matrix with either only one row or one column is referred to as a vector array. You can find more information at this link: MathWorks Matrices and Arrays.

Examples:

1x4 row vector. Use spaces or commas between elements.

- `a = [1 2 3 4];`

4x1 column vector. Use semicolons between rows.

- `b = [1; 8; 2; 6];`

4x3 matrix:

- `c = [1 3 5; 2 4 6; 7 8 10; 9 12 0];`

Array Indexing

To access particular elements of an array, we use indexing. Each element within an array is positioned within a particular row and column; we can use these positions to manipulate specific elements. For more information, see this link: MathWorks Array Indexing

Examples:

Access the element in the 2nd row and 3rd column from matrix c, and assign it to the variable, x.

- `c = [1 3 5; 2 4 6; 7 8 10; 9 12 0];`
- `x = c(2,3);`

Change the value of the element in the 1st row and 1st column of matrix c to 18.

- `c(1,1) = 18;`

Access the entire 3rd row, i.e the 3rd row and all columns, of matrix c and assign it to the variable, x.

- `x = c(3,:);`

Access the entire 2nd column, i.e the 2nd column and all rows, of matrix c and assign it to the variable, y.

- `y = c(:,2);`

Practice working with MATLAB matrices by writing the code below:

7. Assign the ages of patients (HPC1-4) listed in the first column of Table 1 to a new matrix, "ages", with dimensions 1 row and 4 columns (i.e., 1x4 matrix).

```
%Your Code Here. Will require 1 line of code.
```

8. Generate a new 2x4 matrix, "my_data". In the first row, store the age of each patient (measured in years), and in the second row, record the disease chronicity for each patient (also measured in years). Ensure that each column in both rows of the matrix contains data pertaining to the same patient.

```
%Your Code Here. Will require 1 line of code.
```

9. Extract the values in the second row of "my_data".

```
%Your Code Here. Will require 1 line of code.
```

10. Extract the values in the second column of "my_data".

```
%Your Code Here. Will require 1 line of code.
```

```
```

11. Extract the disease chronicity of the patient "HPC1" from "my_data". To answer this, think about which row and column correspond to this value in the matrix "my_data".

```
%Your Code Here. Will require 1 line of code.
```

12. Extract the age of the patient "HPC2" from "my_data".

```
%Your Code Here. Will require 1 line of code.
```

13. Extract the disease chronicity of the patient "HPC3" from "my_data".

```
%Your Code Here. Will require 1 line of code.
```

If the results match those in Table 1 in Spano et al, (2020), you can move to the next section. If they do not, ask your instructor or TA for help.

3. Data Tables

To import a MAT file into MATLAB, follow these steps:

- At the top of the MATLAB window, click the "Home" tab.
- Click the arrow under "Open" and then “Open”
- Navigate to the folder where you downloaded the data file, "Spano2020.mat"
- Select the file "Spano2020.mat" and then click "open" on the bottom right

The MATLAB Workspace should now contain a variable called "Spano2020", a 9x14 table (i.e., 9 rows and 14 columns). Each row represents a specific dream characteristic, and each column corresponds to data for different participants. The first four columns contain data for four patients ('HPC1'. 'HPC2', etc...), while columns five to fourteen store data for ten control participants ('CTL1', 'CTL2', etc..). **To view the row and column names of a table, you can double click the table variable in the MATLAB Workspace.**

"Spano2020" is a table, not a matrix. To extract elements within the table, you can use curly brackets. For example, to retrieve the element in the first row and first column, you can use the syntax:

- `Spano2020{1, 1}`

Alternatively, you can also use the names of the rows and columns you want to select. For instance, to extract the data for the "Number_of_awakenings" characteristic and the "HPC1" participant, you can use:

- `Spano2020{'Number_of_awakenings', 'HPC1'}`

You also have the option to use dot notation, TableName.VariableName(row#), where the variable name is the column name. For example, to extract the dream frequency of the "HPC4" participant, you could use:

- `Spano2020.HPC4(5)`

You can view this link to learn more about Tables: MathWorks Table Access.

You can practice working with MATLAB tables by writing the code below:

14. Extract all the dream data (i.e., all rows) of patient HPC1 from the table "Spano2020" and assign it to the variable, "HPC1_dream". Consider which column contains the data for patient HPC1.

```
%Your Code Here. Will require 1 line of code.
```

15. Extract the dream frequency of all 14 participants (i.e., all columns) from the table "Spano2020" and assign it to the variable "dream_fr". Consider which row contains the dream frequency data.

```
%Your Code Here. Will require 1 line of code.
```

16. Extract the dream frequency of patient HPC1 from the table "Spano2020". Consider which rows and columns include the data of interest.

```
%Your Code Here. Will require 1 line of code.
```

17. Extract the dream frequency of all 4 patients from the table "Spano2020". Consider which rows and columns include the data of interest.

```
%Your Code Here. Will require 1 line of code.
```

If the extracted data match those in Table 2 in Spano et al, (2020), you can move to the next section. If they do not, ask your instructor or TA for help.

4. Data analysis

In this section you will conduct some basic data analysis and learn the following:

- Computing descriptive statistics, including mean, standard deviation, and standard error
- Performing statistical calculations using Mann-Whitney U-test and t-tests
- Carry out within- and between-subject data analysis
- Formulate hypotheses and interpret the results of statistical tests
- Use MATLAB help (To access MATLAB help, click on the question mark button located in the top right corner of the MATLAB window.)

I. Within-subject analysis

It's commonly thought that dreams tend to be more frequent during the REM stage of sleep compared to other stages like NREM. We can empirically test this idea using the data provided by Spano et al. (2020). Please

enter the code below to conduct a within-subject analysis, specifically comparing dream proportions between REM and NREM stages for each participant (including both patients and controls).

18. Extract the data for the proportion of dreams during REM sleep for all fourteen participants from the table "Spano2020" and assign it to a new matrix, "data_REM".

```
%Your Code Here. Will require 1 line of code.
```

19. Compute the average proportion of dreams during REM sleep across all fourteen participants and assign it to the variable, "ave_REM". To do this, use the following MATLAB functions. To compute the mean of matrix "X", the general syntax is "mean_X = mean(X)" where "X" is the input vector and "mean_X" is the output of the function (the mean of "X").

```
%Your Code Here. Will require 1 line of code.
```

20. Compute the standard deviation of the proportion of dreams during REM sleep across all fourteen participants and assign it to the variable, "sd_REM". Use the MATLAB documentation and help menu to look up the function to compute the standard deviation.

```
%Your Code Here. Will require 1 line of code.
```

21. Compute the standard error of the proportion of dreams during REM sleep across all fourteen participants and assign it to the variable, "se_REM". Keep in mind that while the standard error is related to standard deviation, it is not the same. To calculate the standard error, you can divide the standard deviation by the square root of the length of the vector of interest. Use the MATLAB documentation and help menu to look up the function to compute the square root and the vector length.

```
%Your Code Here. Will require ~1 line of code.
```

22. Extract the data for the proportion of dreams during NREM sleep for all fourteen participants from the table "Spano2020" and assign it to a new matrix, "data_NREM"

```
%Your Code Here. Will require 1 line of code.
```

23. Compute the average proportion of dreams during NREM sleep across all fourteen participants and assign it to the variable, "ave_NREM".

```
%Your Code Here. Will require 1 line of code.
```

24. Compute the standard deviation of the proportion of dreams during NREM sleep across all fourteen participants and assign it to the variable, "sd_NREM".

```
%Your Code Here. Will require 1 line of code.
```

```
```

25. Compute the standard error of the proportion of dreams during NREM sleep across all fourteen participants and assign it to the variable, "se_NREM".

```
%Your Code Here. Will require 1 line of code.
```

26. Subtract the average proportion of dreams in NREM from the average proportion of dreams in REM across all participants and assign the difference to the variable, "diff_dreams".

```
%Your Code Here. Will require 1 line of code.
```

27. To assess the statistical significance of the difference in the average proportion of dreams between REM and NREM across participants using a paired sample t-test in MATLAB, you can use the "ttest" function. The general syntax for the "ttest" function is "[~,P] = ttest(X, Y)" where "X" and "Y" are the two matrices you want to compare, and P is the output p-value indicating whether the difference is statistically significant.

In this specific case, you would use "data_REM" as the matrix "X" and "data_NREM" as the matrix "Y" to calculate the p-value (output variable "P"). A "P" < 0.05 suggests a significant difference, while a "P" ≥ 0.05 indicates no significant difference between the groups. Assign your p-value to the variable, "P1".

```
%Your Code Here. Will require 1 line of code.
```

II: Between-subject analysis

Spano et al (2020) hypothesized that the hippocampus plays a critical role in dreaming. Therefore, they predicted that patients with hippocampal damage may experience fewer dreams than healthy controls. In the space below, write the code for a between-subject analysis, specifically comparing dream frequency between hippocampal-damaged patients and healthy controls.

28. Extract the dream frequency of the four patients into a new matrix, "df_HPC".

```
%Your Code Here. Will require 1 line of code.
```

29. Compute the average dream frequency for all patients and assign it to the variable, "df_HPC_ave". In addition, compute both the standard deviation and the standard error and assign these values to the variables, "df_HPC_sd" and "df_HPC_se", respectively.

```
%Your Code Here. Will require 3 line of code.
```

If the results match Table 2 in Spano et al (2020), you can move to the next section. If not, ask the instructor or TA for help.

30. Extract the dream frequency of the ten controls from "Spano2020" into a new matrix, "df_CTL".

```
%Your Code Here. Will require 1 line of code.
```

31. Compute the average dream frequency for all controls and assign it to the variable, "df_CTL_ave". In addition, compute both the standard deviation and the standard error and assign these values to the variables, "df_CTL_sd" and "df_CTL_se", respectively.

```
%Your Code Here. Will require 3 line of code.
```

32. Calculate the difference in average dream frequency between patients and controls by subtracting the average of "df_CTL" from the average of "df_HPC" and assign the difference to the variable, "diff_df".

```
%Your Code Here. Will require 1 line of code.
```

33. To assess the statistical significance of the difference in average dream frequency between patients and controls, you can use the non-parametric Mann-Whitney U test implemented in MATLAB with the "ranksum" function. The general syntax for the "ranksum" function is "P = ranksum(X, Y)," where "X" and "Y" are the two matrices you want to compare, and "P" is the corresponding p-value.

In this specific case, you would use "df_HPC" as the matrix "X" and "df_CTL" as the matrix "Y" to calculate the p-value (variable "P"), which indicates the significance of the difference in dream frequency between the two groups of patients and controls. A "P" < 0.05 suggests a significant difference, while a "P" > 0.05 indicates no significant difference between the groups. Assign your p-value to the variable, "P2".

```
%Your code here. Will require 1 line of code.
```

If the p value indicates a significant difference (similar to what is reported in Table 2), you can move to the next section. Otherwise, ask your instructor or TA for help.

5. Plotting

In the following sections, you will visualize the results of the within- and between-subject analyses using bar plots and learn the following:

- Creating MATLAB matrices for plotting
- Generating Bar Graphs
- Adding error bars, labels for the x and y axes, labels for the ticks on the x axis, and title
- Customizing plots to imrpove data visualization
- Adding comments to MATLAB code
- Saving figures as PNG files

Read and run the following example code to understand how to generate bar charts in MATLAB.

```
%Example code (there is no coding for you to complete here).
MEAN_data = [0.2 0.6]; %  These are the mean values you want to plot.
SEM_data  = [0.01 0.07]; % These are the standard error values associated with the mea
figure % This command creates a new figure window for plotting.
hold all % This command allows you to overlay the bar plot and its corresponding error
bar(MEAN_data) %This line generates a bar plot using the values in the MEAN_data vecto
errorbar(MEAN_data,SEM_data, '.k') %This command adds error bars to the bar plot. The
xticks([1 2]) % This sets the x-axis ticks at positions 1 and 2.
xticklabels({'Group 1', 'Group 2'}) %This labels the x-axis ticks as 'Group 1' and 'Gr
xlabel('x') % This labels the x-axis as 'x'.
ylabel('y') %This labels the y-axis as 'y'.
ylim([0 0.8]) %This sets the limits of the y-axis from 0 to 0.8
title('Example code') %This adds a title to the plot
```

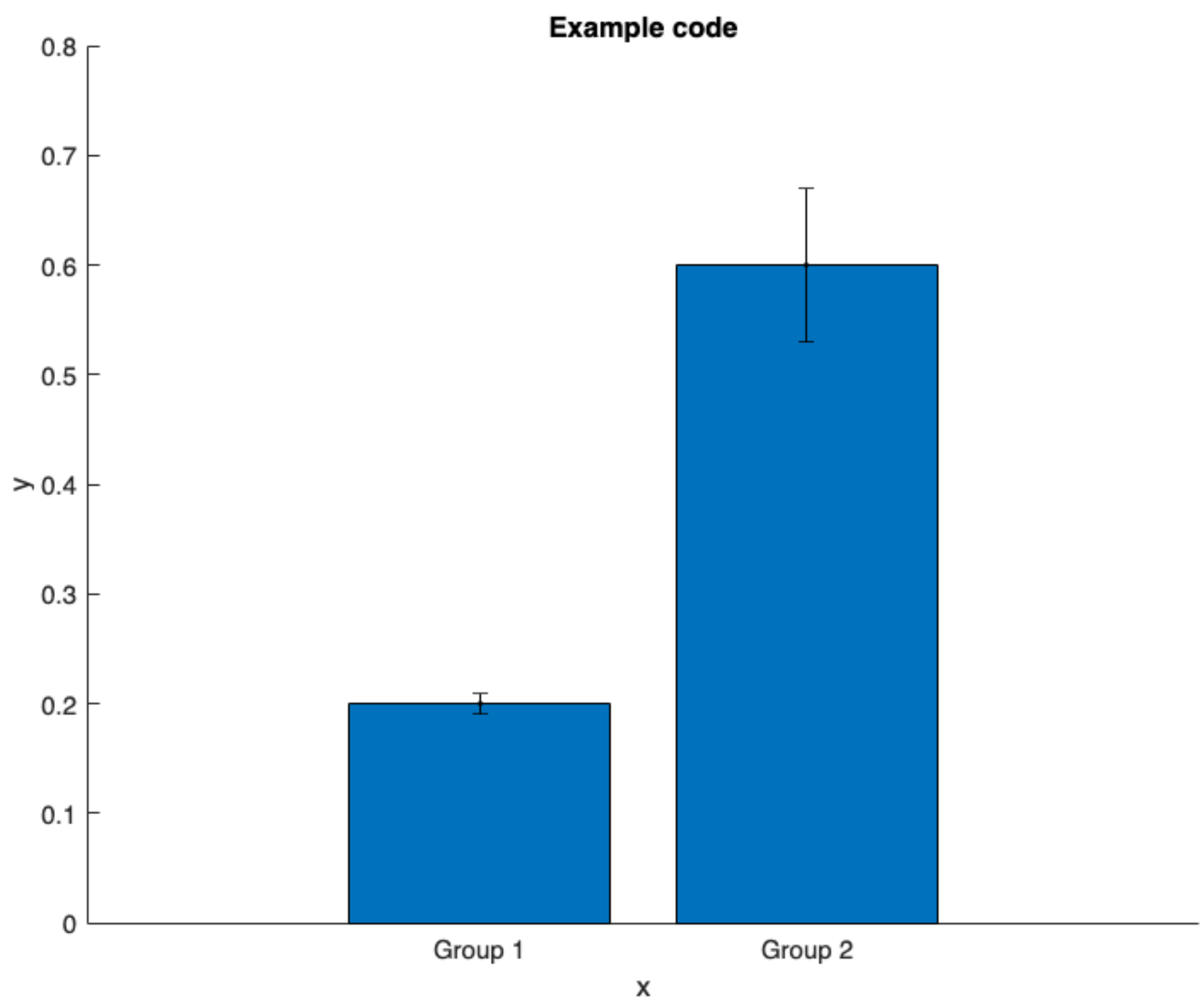

34. Create a 1x2 matrix called "data2plot." In the first column, store the average proportion of dreams during REM sleep ("ave_REM"), and in the second column, store the average proportion of dreams during NREM sleep ("ave_NREM") for all fourteen participants.

```
%Your code here. Will require 1 line of code.
```

35. Create a 1x2 matrix called "SEM_data2plot." In the first column, store the standard error of the proportion of dreams during REM ("se_REM"), and in the second column, store the standard error of the proportion of dreams during NREM ("se_NREM") for all fourteen participants.

```
%Your code here. Will require 1 line of code.
```

36. Based on the example plotting code above, generate a bar chart showing the average proportion of dreams during REM and NREM sleep. Include error bars (standard error), xticks, xticks labels ('REM', and 'NREM'), label of the x-axis ('Sleep stage'), label of the y-axis ('Proportion of Dreams') and title ('Within-subject analysis, REM vs NREM'). This is figure 1.

To customize this plot adjust the y-axis limits to range from 0 to 0.6.

```
%Your code here. Will require ~10 lines of code.
```

37. Based on the example plotting code above, generate a bar chart showing the average dream frequency separately for patients and controls. Include error bars (standard error), xticks, xticks labels ('HPC', and 'CTL'), label of the x-axis ('Groups'), label of the y-axis ('Dream frequency') and title ('Between-subject analysis, dream frequency'). This is figure 2.

To customize this plot, adjust the y-axis limits to range from 0 to 1 and set the bar color to magenta. Refer to the MATLAB documentation regarding the function "bar()" for guidance on how to do this.

Use "%" to make brief comments describing each line of your code.

```
%Your code here. Will require ~12 lines of code
```

If your figure 2 matches Figure 1 panel D in Spano et al, (2020), you can move to the next section. If they do not, ask your instructor or TA for help.

5. Saving MATLAB figures and code

In this section you will learn the following:

- Saving MATLAB figures in PNG format
- Saving MATLAB code

38. To save MATLAB figures, use the "Save as" button on the top left of the figure window. Choose the PNG format. You should save the first and second figure you created with titles "Figure1.png" and "Figure2.png", respectively.

If you can't see the figure window, hover over the top right corner of the figure in the live Editor and click "Open in figure window".

39. To save your MATLAB code, use the "Save" button on the top left of the MATLAB window. Name it “w5_matlabcode_lastname.mlx”, e.g. "w5_matlabcode_alexandrescu.mlx".

40. S*ubmit your MATLAB code in MLX format.*

***6.* Questions:** Answer the questions below based on the figures and statistical results you obtained.

Q1: Submit your MATLAB Figure 1. Write a caption for Figure 1. The caption should include a descriptive title, a brief description of materials and methods, a brief description of the results, descriptive statistic(s) shown, statistical test(s) used, p-value(s), sample size (n) for all groups, explanations of figure features (e.g., symbols, lines, colors, error bars, etc.). Consult the listed websites to find out what should be included in a scientific figure caption/legend. You can also view examples from published papers on Google Scholar.

- https://www.aje.com/arc/writing-effective-figure-legend/
- https://www.scu.edu/media/offices/provost/writing-center/resources/Tips-Figure-Captions.pdf

Q2: Submit your MATLAB Figure 2. Write a caption for Figure 2.

Q3: Based on this data analysis, does dreaming occur more often during REM sleep compared to NREM sleep? Explain based on the average data and p-value you calculated and refer to the null hypothesis in your explanation. Use no more than 1-3 sentences.

Q4: Based on this data analysis, do patients with hippocampal damage experience fewer dreams? Explain based on the average data and p-value you calculated and refer to the null hypothesis in your explanation. Use no more than 1-3 sentences.
