# Supplementary material for: “I Can Code”: An Innovative Workshop Introducing Students to Coding in the Context of Neuroscience Research
Source: J Undergrad Neurosci Educ. 2026 May 19;24(2):51–60. doi: 10.59390/001c.160812 (PMC13625906; doi:10.59390/001c.160812)
Supplement: Supplementary file 2 [file junejournal_2026_24_2_160812_340612.pdf]

1. Identify the independent variable (IV) and the dependent variable (DV) in the following hypothesis:

Sleep-deprived college students perform worse on concentration tasks than rested college students.

IV: amount of sleep; DV: performance on concentration tasks

IV: performance on concentration tasks; DV: amount of sleep

IV: college students; DV: amount of sleep

IV: sleep-deprived college students; DV: rested college students

2. Identify the independent variable (IV) and the dependent variable (DV) in the following hypothesis:

When an individual is exposed to an unexpected loud noise, their blood adrenaline level will increase within minutes.

IV: unexpected loud noise; DV: no noise

IV: blood adrenaline level; DV: exposure to an unexpected loud noise

IV: minutes; DV: blood adrenaline level

IV: exposure to an unexpected loud noise; DV: blood adrenaline level

3. In a research study determining if attending recitations improves exam scores among college students, which of the following is most likely to be a confounding variable?

the day of the week the exam is taken

the color of notebooks used by the student

the number of hours a student studies for the exam

the brand of laptop used by the student

4. Identify the measurement scale for the following type of data:

years (e.g., 1998, 2024)

nominal

ordinal

interval

ratio

5. Identify the measurement scale for the following type of data:

academic majors

- **nominal**
- ordinal
- interval
- ratio

6. Identify the measurement scale for the following type of data:

weight

- nominal
- ordinal
- interval
- **ratio**

7. Identify the measurement scale for the following type of data:

student ratings of their satisfaction level with the cafeteria menu on a scale from 1 to 5, with 1 being "Very Unsatisfied" and 5 being "Very Satisfied"

- nominal
- **ordinal**
- interval
- ratio

8. Identify the measurement scale for the following type of data:

the number of pyramidal neurons in a specific brain region

- nominal
- ordinal
- interval
- **ratio**

9. Identify the measurement scale for the dependent variable in the following example:

In a survey about the effect of caffeine on sleep habits, students are asked whether they consider themselves a "Night Owl", "Early Bird", or "Neither".

nominal

ordinal
interval
ratio

10. In an experiment investigating the effects of sleep deprivation on cognitive performance, participants were divided into one of three groups:

Group A: Had a normal night's sleep

Group B: Had 4 hours of sleep

Group C: Had no sleep

Answer questions 10-11 based on the information provided above.

Which group was the control group?

Group A

Group B
Group C
There was no control group

11. What type of subject design was implemented in the study above?

between-subjects design

within-subjects design

12. In another experiment examining the effects of auditory stimulation on memory retention, each participant was exposed to all of three conditions while memorizing a list of words:

Condition A: Listening to classical music

Condition B: Listening to white noise

Condition C: Listening to silence

Answer questions 12-13 based on the information provided above.

How many levels of the independent variable were used in this experiment? Type a whole number in the space below.

3.00

13. Considering all participants went through all conditions at different times, what type of subject design was implemented in the study above?

between-subjects design

within-subjects design

14. The __________ is the value that appears most frequently in a dataset.

mean

median

mode

15. The __________ is the middle value in a dataset when the numbers are arranged in an ascending or descending order. If there is an even number of values, it is the average of the two middle numbers.

mean

median

mode

16. If the variance of a dataset increases:

the standard deviation and the standard error of the mean increase

the standard deviation increases and the standard error of the mean decreases
the standard deviation decreases and the standard error of the mean increases
the standard deviation and the standard error of the mean decrease

17. If the graph depicting the distribution of values in a dataset has a bell shape, the distribution is said to be:

a normal distribution

an average distribution
a natural distribution
a common distribution

18. Consider the following hypothetical experiment and answer questions 18 - 21 based on the information provided below.

You design a research study to investigate the effects of note-taking techniques (hand-written vs. typed on a laptop) on the ability to retain information in two different groups of college students.

What is the null hypothesis in your study?

The type of note-taking technique has no effect on the students' ability to retain information.

The type of note-taking technique affects the students' ability to retain information.

19. What is the alternative hypothesis in your study?

The type of note-taking technique has no effect on the students' ability to retain information.

The type of note-taking technique affects the students' ability to retain information.

20. What type of parametric statistical test would you use to help you decide between these two competing explanations (i.e., null vs. alternative hypotheses) of any differences observed between the groups in your study?

Unpaired t-test

Paired t-test
Ordinary one-way ANOVA
Repeated measures one-way ANOVA
Mann-Whitney U test

21. If the result of your statistical test produced a *p*-value of less than 0.01, how would you interpret it? Select ALL that apply.

the observed difference between the 2 groups is most likely due to random variability

the observed difference between the 2 groups is most likely due to the type of note-taking technique
the result is statistically significant
you can reject the alternative hypothesis in favor of the null hypothesis
you can reject the null hypothesis in favor of the alternative hypothesis
the probability that the difference between the 2 groups is due to the note-taking technique is more than 99%
the result proves that the alternative hypothesis is true

22. Answer the following questions based on the paper by Spano et al. (2020).

Which of the following best represents the main hypothesis in the study?

individuals with damaged hippocampi tend to experience more vivid dreams than those with intact hippocampi

participants with hippocampal damage will wake up more frequently during the night than control participants
the hippocampus has no significant role in the episodic-like nature of dreams
patients with hippocampal damage will exhibit reduced frequency and altered content of dreams compared to control participants

23. What is the independent variable in the study?

the number of times participants were awakened during the night

the frequency of dreams reported by the participants

the presence or absence of hippocampal damage in the participants

the episodic-like nature of participants' dreams

24. What is the dependent variable in the study?

the frequency and episodic-like nature of dreams reported by the participants

the presence or absence of hippocampal damage in the participants

the number of times participants were awakened during the night

the presence or absence of amnesia in the participants

25. Which of the following is an extraneous variable the researchers controlled for in the study?

the presence or absence of hippocampal damage in the participants

the number of times participants were awakened during the night

the frequency of dreams reported by the participants

whether the participants enjoy dreaming

26. Identify the measurement scale for the following type of data from the study:

right-handedness of participants

nominal

ordinal

interval

ratio

27. Identify the measurement scale for the following type of data from the study:

the number of times participants were awakened during the night

nominal

ordinal

interval

ratio

28. Identify the measurement scale for the following type of data from the study: proportion of blank dreams reported by participants

nominal

ordinal

interval

ratio

29. Identify the measurement scale for the following type of data from the study: hippocampal damage chronicity (years) in patients

nominal

ordinal

interval

ratio

30. How many conditions were used in the study?

one

two

three

four

31. What type of subject design was used in the study to test the main hypothesis?

between-subjects design

within-subjects design

32. How many participants were in the experimental group?

1

4

6

10

33. What is the volume (in mm^3) of the right hippocampus of patient HPC1? To answer this, inspect the demographic characteristics in Table 1.

2506

2803

3285.91

1698

34. How many times did patient HPC2 wake up during the night? To answer this, inspect the data reported in Table 2.

6.75

6.90

10.00

4.00

35. What method was used to differentiate between NREM and REM sleep?

Voxel-based morphometry with MRI

Polysomnography (PSG) with EEG

The Scene Construction test by Hassabis et al. (2007)

Mann-Whitney U test

36. What method was used to measure hippocampal volume?

Structural MRI

Functional MRI

EEG

SPSS

37. What method was used to collect data on participants' dreams and their content?

A post-sleep questionnaire asking participants to recall their dreams the morning after

Continuous monitoring of various physiological parameters such as brain activity, eye movements, and heart rate

Machine learning algorithms applied to continuous recordings of brain activity during sleep

A protocol that periodically woke participants during the night to report their thoughts (i.e., serial awakening paradigm)

38. For which of the following dependent variables did the authors report a statistically significant difference between the patients and control participants?

age of participants

number of awakenings

dream frequency

IQ of participants

39. Why do you think the authors used a Mann-Whitney U test to determine if there was a statistically significant difference in dream frequency between patients and control participants?

the data points showed a non-normal distribution

the data points were measured on a ratio scale

the data points showed a bell-shaped distribution

it is a commonly used parametric test

40. What is one of the key conclusions drawn from the study's results regarding the relationship between hippocampus and dreaming?

dreaming plays an important role in supporting memory consolidation in patients with hippocampal damage

memory processing relies on the structure and function of the hippocampus

hippocampal integrity is critical for dreaming

dreaming is correlated with activity in the hippocampus in both patients and healthy participants
