# Supplementary material for: “I Can Code”: An Innovative Workshop Introducing Students to Coding in the Context of Neuroscience Research
Source: J Undergrad Neurosci Educ. 2026 May 19;24(2):51–60. doi: 10.59390/001c.160812 (PMC13625906; doi:10.59390/001c.160812)
Supplement: Supplementary file 3 [file junejournal_2026_24_2_160812_340613.pdf]

EXPERIMENTAL DESIGN AND STATISTICS GUIDE

In this course, you will propose and test experimental hypotheses related to brain function and behavior. For instance, you'll explore questions and predictions related to the role of a brain region like the hippocampus in a specific behavior like dreaming. You will also learn how to analyze anatomical data, such as comparing the size of a brain structure across hemispheres. To evaluate these effects, it is necessary to understand and use **experimental design** to devise, conduct, and analyze research studies. Within these experiments you will use manipulations that may cause changes in the brain or in behavior, for example, 'hippocampal damage reduces dream frequency', or 'time of day regulates neurotransmitter release'. How do we know whether any specific change we observe in the laboratory is real as opposed to random variation? That is, how reliable is the change, how great is the change, and is the change due to the manipulation we made or some other factor? To answer these questions, it is necessary to use **methods of statistics**. This guide will introduce you to experimental design concepts and statistical techniques prevalent in neuroscience research.

EXPERIMENTAL VARIABLES

In experimental design, understanding and categorizing variables is crucial for ensuring the validity and reliability of your research outcomes. A variable is any factor that can vary (i.e., take more than one value) in an experiment. There are three types of experimental variables: independent, dependent, and extraneous.

Imagine you are a student at Barnard College, sipping your coffee at Liz's Place. You wonder whether the temperature of your coffee affects how much you enjoy it. In this scenario, the temperature of the coffee (which can be adjusted) is the **independent variable**, because you believe it may *influence* the outcome (i.e., how much you enjoy it). Your level of enjoyment, which you measure by ranking it on a scale from 1 to 5, is the **dependent variable**, as it potentially *depends* on the coffee's temperature. All other factors, like the brand of coffee, the amount of sugar or milk added, or the ambient noise level in Liz's Place, would be **extraneous variables**, as they might influence your coffee enjoyment. But what if you tend to drink hotter coffee when you're in a particularly good mood? This would make it difficult to determine if your enjoyment is due to the coffee temperature or your good mood. This is an example of a **confounding variable**, a type of extraneous variable that is associated with both the independent and dependent variables. To make sure only the coffee temperature affects your enjoyment, it's crucial to keep extraneous variables consistent. Much like this coffee scenario, in neuroscience experiments, identifying and correctly handling experimental variables is essential to draw meaningful conclusions.

Independent Variable: the variable that is deliberately manipulated in an experiment to observe its possible effects on the dependent variable
Dependent Variable: the variable that is observed or measured in an experiment, which may change as a result of the independent variable's manipulation
Extraneous Variable: any variable other than the independent variable that could influence the dependent variable if not controlled
Confounding Variable: a type of extraneous variable that is correlated with both the independent and dependent variables, hence directly impacting the cause-and-effect relationship between them and confounding the interpretation of the experiment
Controlled Variable: any variable that is kept constant throughout the experiment to ensure that any observed changes in the dependent variable are solely due to the manipulation of the independent variable

MEASUREMENT SCALES OF EXPERIMENTAL VARIABLES

Experimental variables can also be categorized based on the type of data they produce, dictated by their measurement scale: **nominal, ordinal, interval, and ratio** (NOIR). The table below lists the characteristics and examples of data that are measured using different scales. Recognizing the measurement scale of variables is important, as it dictates the appropriate statistical analyses and determines the type of insights one can infer from the data.

Table 1. Measurement Scales

**Scale**	**Characteristics**	**Examples**
Nominal	Categorized data<br>*No meaningful order, intervals, or zero point*	Color<br>Brand<br>Type of stimulus (visual, auditory)
Ordinal	Categorized and ordered data<br>*No equal intervals between categories*<br>*Arbitrary or absent zero point*	Rankings<br>Clothing sizes<br>Likert scale
Interval	Categorized and ordered data<br>Equal intervals between categories<br>*Arbitrary or absent zero point*	Temperature (Celsius/Fahrenheit)<br>Time of day<br>IQ
Ratio	Categorized and ordered data<br>Equal intervals between categories<br>*True zero point	Temperature (Kelvin)<br>Height<br>Number of correct answers

* The true (absolute) zero point represents the absence of the variable being measured.

NUMBER OF CONDITIONS IN AN EXPERIMENT

When structuring an experiment, understanding the number of conditions (i.e., the levels of the independent variable) is paramount, as it shapes the depth and breadth of information gleaned from the study. Most experiments will have at least two conditions or groups: a control group and an experimental group. The **control group** provides a baseline, receiving no treatment or manipulation, whereas the **experimental group** undergoes the manipulation or intervention. For instance, in a clinical study exploring a new drug's effect on memory, the experimental group receives the drug, while the control group receives a placebo (e.g., a sugar pill). However, many experiments opt for more than just these two groups to examine varying degrees or types of interventions. In the example above, additional experimental groups that receive different doses of the drug might be added. Including multiple levels of the independent variable in an experiment allows researchers to capture not just whether an independent variable has an effect, but also how variations in that variable (e.g., different doses, time points) might lead to different outcomes.

SUBJECT EXPERIMENTAL DESIGN

Experimenters have multiple approaches to assigning subjects to different experimental conditions and the choice between them can dramatically affect the results and interpretations of the experiment. There are primarily two types of subject designs: between-subjects and within-subjects.

A **between-subjects design**, also known as an independent measures design, involves assigning each subject to only one condition in an experiment. This means different groups of subjects experience different levels of the independent variable. For instance, in a neuroscience experiment testing the effects of two different diets, a low and a high-caloric one, on cognitive performance, one group of rats might be fed the low-caloric diet while another group might receive the high-caloric diet. This approach mitigates the risk of order effects or carry-over effects from one condition to another. However, it requires more subjects and can be vulnerable to individual differences influencing outcomes. One way to minimize possible confounding effects of individual differences is to randomly assign participant to conditions.

Conversely, in a **within-subjects design**, also known as a repeated measures design, each subject experiences all levels of the independent variable. If the same diet experiment was conducted using a within-subjects design, each rat would undergo cognitive assessment after being fed the low-caloric diet and again after being fed the high-caloric diet, though not necessarily in that order. This design requires fewer subjects and controls for individual differences, but it might introduce order effects (e.g., carry-over, practice, fatigue), where the sequence of conditions affects the results. One way to prevent these order effects from becoming confounding is counterbalancing the order of conditions.

STATISTICAL METHODS

Statistical methods that allow us to describe our data in concise mathematical terms are referred to as **descriptive statistics.** Similarly, we use well-established norms for determining the significance of a finding in the statistical method referred to as **inferential statistics**.

DESCRIPTIVE STATISTICS

Descriptive statistics provide a summary and insights into your data, simplifying large amounts of information into more digestible values or figures. This form of statistics plays a crucial role in the preliminary understanding of experimental data before inferential analyses are performed.

MEASURES OF CENTRAL TENDENCY

The **central tendency** of a data set is a single number that characterizes the data by providing information about its "middle" value. There are three commonly used measures of central tendency: mean, median, and mode.

Consider the following data set consisting of test scores arranged in ascending order:

48 53 59 64 65 74 74 78 81.5 83 85 85 85 87.5 89 91 93 95

The **mean** of this distribution is the sum (Σ) of all test scores divided by the number (n) of test scores (77.2).
The **median** is the mid-point of the distribution, the score that divides the distribution into equal halves (when the data points are arranged in ascending or descending order). If there is an even number of values, it is the average of the two middle numbers. In the above example, there are 18 scores; therefore, the mid-point occurs between the 9th and 10th scores (82.25).
The **mode** of this distribution is the score that occurs most frequently (85).

MEASURES OF VARIABILITY

The variability describes the amount of variation among the values in the data set. If the scores are tightly clustered around the mean, then variability is low; if they are widely scattered, the variability is high. One quick way to describe variability is to calculate the **range** of the data (highest score minus lowest score). In the example given above, the range is 47 (95 minus 48). If the data were instead 2 scores of 2 and 16 scores of 85, the range would be 83 (85 minus 2).

The range does not give us all the information we need, however; a more complete description of the variability can be achieved by calculating the **variance**, or a related measure called the **standard deviation**. These measures are based on the difference between each score in the data set and the mean value and therefore describe the dispersion of the data around the mean. To calculate the variance of a data set, we take the difference between each data point (x) and the mean ($\bar{x}$), square each difference (so the result is positive), add all the squared differences up, and divide by n-1 (where n is the number of data points in the data set); the standard deviation (SD) of a sample is simply the square root of the variance:

$$\text{SD} = \sqrt{\frac{\Sigma(x-\bar{x})^2}{n-1}}$$

For the data set given above, the standard deviation is 13.3. If we consider the second case, where the data were 2 scores of 2 and 16 scores of 85, the mean would still be similar (75.7), but the standard deviation would be much larger (26.8).

The **standard error of the mean** (SEM) indicates the variability of the mean if we were to conduct the same experiment multiple times and is calculated simply by taking the standard deviation and dividing it by the square root of the sample size. For our test score example, given our SD of 13.3 and sample size of 18, the SEM is 3.1, while for the second set of scores with an SD of 26.8, it is 6.3.

$$\text{SEM} = \frac{\text{SD}}{\sqrt{n}}$$

When the number of data points is large (e.g., >30), their distribution approximates a theoretical curve called a **normal distribution**, often referred to as a *bell curve* because of its characteristic bell-shaped graph. For a normal distribution, 67% of the data points are contained within a range of one standard deviation above and below the mean, and 93% of the data points are contained within two standard deviations from the mean. The figure below shows the comparison of two normal distributions with the same number of data points and the same mean but with different values of standard deviation. In one case the standard deviation is small, so the distribution appears as a tall and narrow peak. This reflects the fact that the majority of the data points are very close to the mean and there is little variability in the sample. In the other case, however, the distribution is short and spread out since only a small number of the data points are close to the mean, which produces a large standard deviation.

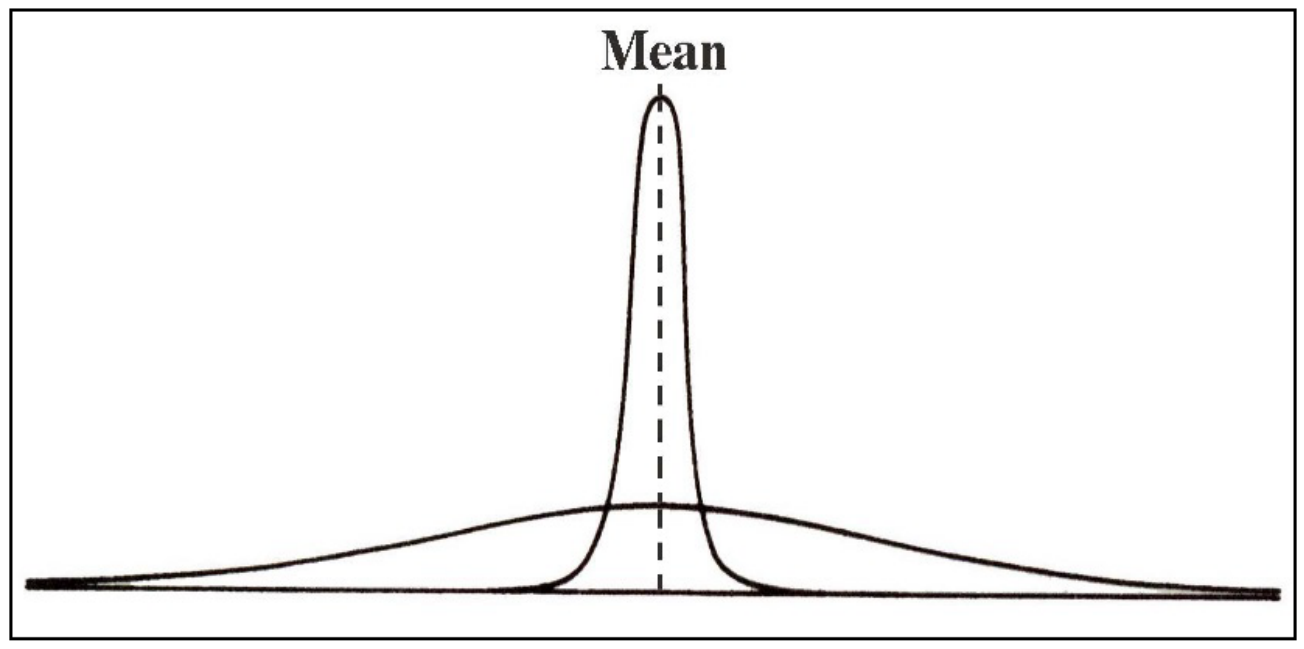

INFERENTIAL STATISTICS

In addition to using statistics to describe large collections of numerical information, we also use statistical methods to draw inferences from the results of experiments. We don't simply want to report the scores of two groups of subjects or give the mean and standard deviation and leave it at that. We want to use the data to test a prediction or **hypothesis** - to decide whether the independent variable is having the effect we supposed, or whether, perhaps, there is no real difference between the performance of the two groups. This is the function of **inferential statistics**.

STATISTICAL SIGNIFICANCE

We can represent the process of statistical inference as deciding between two competing explanations of the difference(s) between different sets of data points:
The **null hypothesis (H_0)**: the differences arise because of random variability in the data sets.
The **alternative hypothesis (H_1)**: the differences are caused, at least in part, by the independent variable. The alternative hypothesis corresponds to the experimenter's prediction and is sometimes called the experimental hypothesis.

Referring back to our coffee scenario, the null hypothesis states that there is no relationship between coffee temperature and level of enjoyment, while the alternative hypothesis affirms that the temperature of the coffee does affect enjoyment.

Using this terminology, we can now say that a statistical test tells us the probability that the results could have occurred *under the null hypothesis* (i.e., purely by chance). This probability is referred to as the ***p*-value**. The smaller the *p*-value, the stronger the evidence against the null hypothesis, leading researchers to consider it less likely to be true. The probability of an event is the likelihood that it will occur, expressed on a scale from 0 to 1, where 0 represents no chance of it occurring and 1 means that it is certain to occur. In many fields, including neuroscience, a probability threshold, often set at 0.05 is employed: if the *p*-value is less than this value, the results are deemed "*statistically significant*", and the null hypothesis is rejected in favor of the

alternative hypothesis (i.e., the probability that the difference between the sets of scores was due to chance is less than 5%).

It is crucial to understand, however, that a small *p*-value (e.g., <0.5) does not confirm the truth of the alternative hypothesis; it merely suggests that the observed data is inconsistent with what we would expect under the null hypothesis. Likewise, a large p-value (e.g., >0.5) does not confirm the truth of the null hypothesis; it tells us that we were unable to reject it. This is because hypotheses can never be proven true. At the most, they can be falsified.

STATISTICAL TESTS

There are two major factors that determine the choice of statistical test for any particular set of experimental results: (1) the experimental design (e.g., between/within-subjects design, number of conditions) and (2) the nature of the dependent variable, that is, the actual data. There are two types of statistical tests - parametric and non-parametric.

Parametric tests are based on highly restrictive assumptions about the type of data that are obtained in the experiment: (1) sample scores have been drawn from populations that have normal distributions; (2) these populations have the same variance; (3) the dependent variable has been measured on an interval or ratio scale. Because of these assumptions, parametric tests can be more powerful (i.e., more likely to detect a true effect if one exists) when the assumptions hold true. However, they can be misleading if these assumptions are violated.

Non-parametric tests, on the other hand, do not make strong assumptions about the data's distribution (i.e., normality and variability) and can be used with nominal, ordinal, interval, or ratio data. While they are more robust against variations of assumptions, they may be less powerful than parametric tests when the data are normally distributed.

Let's expand on the coffee scenario with some examples illustrating when you might choose a parametric versus a non-parametric statistical test to assess the hypothesis that coffee temperature influences the level of enjoyment:

Example 1: Parametric Test

You decide to use a method that provides a more objective measurement than self-report scales to evaluate coffee enjoyment. Using fMRI (functional Magnetic Resonance Imaging) you assess coffee enjoyment by measuring the level of activation (from a baseline level before coffee drinking starts) of regions of the brain associated with pleasure and reward. After gathering data from several fellow students assigned to one of two conditions (two different coffee temperatures), you find that their fMRI measurements, which are measured on a ratio scale, have a bell-shaped or normal distribution and have similar variance. Since your data meets the assumptions for parametric tests, you decide to use a parametric test, like the

unpaired *t*-test, to determine if there is a statistically significant difference in enjoyment between two different temperatures.

Example 2: Non-parametric Test

After gathering data from two experimental groups given two different coffee temperatures, you realize that the enjoyment scores, which you measured with a self-report scale of 1 to 5, are not normally distributed; perhaps most students gave extreme scores (either 1s or 5s) with few in the middle. Given the non-normal distribution and the ordinal scale of measurement of your enjoyment scores, you opt for a non-parametric test, such as the Mann-Whitney U test, to analyze your data.

Below is a table summarizing common statistical tests used in neuroscience and the experimental designs in which they are typically used.

Table 2. Commonly Used Statistical Tests

**Parametric test**	**Non-parametric test**	**Purpose of test**	**Example**
Independent samples (unpaired) *t*-test	Mann-Whitney U test	Compares the means of two independent groups (between-subjects design)	Compare the mean exam scores of students in morning vs. evening classes
Related samples (paired) *t*-test	Wilcoxon signed-rank test	Compares the means of two related groups (within-subjects design)	Compare the mean exam scores of the same group of students on vs. off caffeine
Ordinary one-way analysis of variance (ANOVA)	Kruskal-Wallis test	Compares the means of three or more independent groups based on one independent variable	Compare the mean sleep hours of students in different years of study
Repeated measures one-way analysis of variance (ANOVA)	Friedman test	Compares the means of three or more related groups based on one independent variable	Compare the mean sleep hours of the same group of students in 3 different semesters
Pearson's *r* correlation coefficient	Spearman rank test	Quantifies the linear relationship between two variables	Correlate the number of hours spent studying with GPA
